# Review of gastrointestinal bleeding during use of SSRIs combined with use of NSAID

**DOI:** 10.1192/j.eurpsy.2022.439

**Published:** 2022-09-01

**Authors:** M. Arts, S. Petrykiv, L. De Jonge

**Affiliations:** 1 GGZWNB, Psychiatry, Halsteren, Netherlands; 2 Leonardo Scientific Research Institute, Neuropsychiatry, Halsteren, Netherlands

**Keywords:** bleeding, SSRIs, NSAID

## Abstract

**Introduction:**

In recent years, more and more attention has been paid to the risks of using SSRIs. This group of antidepressants may be associated with an increased risk of gastrointestinal bleeding. This risk would be even further increased with concomitant use of NSAIDs. A number of studies have described this interaction, however they reported conflicting results.

**Objectives:**

Our objective was to investigate the risk of gastrointestinal bleeding with SSRIs, with or without NSAID use.

**Methods:**

We performed a literature search, using Pubmed, EMBASE, and Cochrane library, in order to investigate controlled trials, cohort, case-control and cross-sectional studies that reported the incidence of gastrointestinal bleeding s on SSRIs with or without concurrent NSAID use, compared to placebo or no treatment.

**Results:**

15 case-control studies and 4 cohort studies were included in the analysis. There was an increased risk of gastrointestinal bleeding with SSRIs in the cohort studies and case-control studies. The risk of gastrointestinal bleeding was even further increased with the combined use of both SSRIs and NSAIDs.

**Conclusions:**

SSRIs are associated with a modest increase of gastrointestinal bleeding. However, this risk is significantly increased when SSRIs are used in combination with NSAIDs. Psychiatrists should be aware of the hazards in prescribing these medications together.

**Disclosure:**

No significant relationships.

